# When Eosinophilic Granulomatosis With Polyangiitis (EGPA) Looks Like Granulomatosis With Polyangiitis (GPA): Navigating a Complex Case of Anti-neutrophil Cytoplasmic Antibodies (ANCA) Vasculitis

**DOI:** 10.7759/cureus.116190

**Published:** 2026-09-13

**Authors:** Monica Kontor, Raheem Robertson, Nkechi Ukoha, Joan Morny, Ashley Thompson-Edwards

**Affiliations:** 1 Internal Medicine, Piedmont Athens Regional Medical Center, Athens, USA

**Keywords:** anca-associated vasculitis, anca pr-3 antibodies, biologic agents, case report, corticosteroid, eosinophilic granulomatosis with polyangiitis (egpa), granulomatosis with polyangiitis (gpa), immunofluorescence, overlap syndromes, systemic vasculitis

## Abstract

Eosinophilic granulomatosis with polyangiitis (EGPA) is an anti-neutrophil cytoplasmic antibody (ANCA)-associated vasculitis, with myeloperoxidase antibodies in 40% of cases. Proteinase 3 (PR3) positivity is rare and may overlap with granulomatosis with polyangiitis (GPA). We present a 43-year-old male with recurrent sinusitis, epistaxis, neuropathic pain, and acute kidney injury, with histopathology of kidney biopsy showing EGPA despite the absence of asthma, which is rare. His PR3 positivity and clinical presentation mimicked GPA, highlighting the diagnostic challenges of vasculitides. We report this case to highlight that EGPA can have an atypical presentation. PR3-ANCA positivity and absence of asthma do not exclude EGPA. Also, renal biopsy is crucial for diagnosis when serology and clinical features do not align.

## Introduction

Eosinophilic granulomatosis with polyangiitis (EGPA), historically known as Churg-Strauss syndrome, and granulomatosis with polyangiitis (GPA), historically known as Wegener's granulomatosis, are both systemic vasculitides with overlapping clinical features, making accurate diagnosis challenging [1]. Despite the rarity of EGPA, with a global prevalence of 2.0 to 38.0 per million people, the range of organ systems involved and the relapsing-remitting nature of the disease exert a large burden on both patients and healthcare systems [2]. The 2022 American College of Rheumatology/European Alliance of Associations for Rheumatology (ACR/EULAR) classification criteria provide current criteria for anti-neutrophil cytoplasmic antibody (ANCA)-associated vasculitides. For EGPA, a score of ≥6 points is required for classification, with marked blood eosinophilia, obstructive airway disease, nasal polyps, and eosinophilic tissue inflammation being important positive features. For GPA, a score of ≥5 points is required, with proteinase 3 (PR3)-ANCA positivity, sinonasal disease, pulmonary nodules or cavitation, and granulomatous inflammation contributing positively to classification [3]. These criteria are intended for classification rather than establishing the clinical diagnosis. This case report explores the complexities of distinguishing between EGPA and GPA, highlighting the need for a thorough understanding of their clinical presentations, diagnostic criteria, and appropriate laboratory investigations.

We present a case of a 43-year-old male with a history of recurrent sinusitis with recurrent epistaxis, neuropathic pain in the legs, and no medical history of asthma, who presents with PR3 antibody-positive EGPA.

## Case presentation

A 43-year-old male with a history of recurrent sinusitis, recurrent epistaxis, and neuropathic leg pain, but no history of asthma, presented to the emergency department with epistaxis, abdominal pain, and rectal bleeding. He had multiple episodes of sinusitis and epistaxis for about six months before presentation. He had neuropathic pain in both lower extremities for about a year. The patient had no history of previous medical conditions and did not take any medications. He did not smoke, drink alcohol, or use recreational drugs. He had a family history of diabetes in his maternal grandfather; no other pertinent family history. One month before this presentation, he was hospitalized for fever, chills, shortness of breath, pleuritic chest pain, and generalized body aches, suspected to be sepsis, but infectious workup was negative. Eosinophilia and renal impairment were not present at the time. On presentation, he was tachycardic (127 bpm), febrile (102°F), and hypotensive (90/75 mmHg). Physical examination revealed crusty nasal discharge without active epistaxis, clear lungs, and mild left lower quadrant abdominal tenderness. Initial laboratory findings shown in Table 1 included leukocytosis (31.6 x 10^9^/L), low hemoglobin (6.5 g/dL), eosinophilia (2.5 x 10^9^/L), acute kidney injury (creatinine: 2.03 mg/dL), transaminitis (aspartate aminotransferase: 169 u/L, alanine aminotransferase: 162 u/L), and significant hematuria (RBCs: 80/hpf) with 2+ proteinuria. Imaging, including abdominal CT and magnetic resonance cholangiopancreatography, was unremarkable. Despite supportive management, his creatinine continued to rise, peaking at 6.7 mg/dL. Nephrology was consulted, and hemodialysis was initiated. Infectious workup was negative, and autoimmune screen revealed a positive C-ANCA, elevated PR3 antibodies (384 AI, normal <1 AI), and slightly elevated rheumatoid factor at 51 IU/mL, with low complement C3 and C4 levels. While on admission, the patient continued to have epistaxis, for which he required cauterization. Also regarding his neuropathic leg pain, he subsequently had nerve conduction studies of the bilateral lower extremities, which were consistent with mild right-sided involvement and asymmetrically severe left-sided involvement involving multiple nerves, both sensory and motor. It, however, did not reveal evidence of lumbar radiculopathy in the left lower extremity of the muscles that were tested, as there was no evidence of denervation or re-innervation potentials. This was suggestive of mononeuritis multiplex.

**Table 1:** Laboratory results during admission Highlighting eosinophilia (2.5 x 10⁹/L), acute kidney injury (creatinine elevated at 2.03 from baseline of 1.1 mg/dL), leukocytosis (31.6 x 10⁹/L), low hemoglobin (6.5 g/dL), transaminitis (AST: 169 u/L, ALT: 162 u/L), significant hematuria (RBCs: 80/hpf) with 2+ proteinuria, autoimmune screen with a positive C-ANCA, elevated proteinase 3 antibodies (384 AI, normal <1 AI), low complement levels (C3: 75, C4: <8 mg/dL), and slightly elevated rheumatoid factor at 51 IU/mL. ALT, alanine aminotransferase; ANA, antinuclear antibody; ANCA, antineutrophil cytoplasmic antibody; ASL, antistreptolysin; BUN, blood urea nitrogen; C-ANCA, cytoplasmic antineutrophil cytoplasmic antibody.

Test	Value	Reference range
Sodium	139	137-145 mmol/L
Potassium	3.7	3.5-5.1 mmol/L
Chloride	98	98-107 mmol/L
Bicarbonate	35	22-30 mmol/L
Anion gap	10	10-20
BUN	49	9-20 mg/dL
Creatinine	2.03	0.66-1.0 mg/dL
Glucose	136	74-100 mg/dL
Calcium	8.5	8.4-10.2 mg/dL
Alkaline phosphatase	75	38-126 U/L
Albumin	3.1	3.5-5.0 g/dL
Total protein	5.6	6.3-8.2 g/dL
AST	169	17-59 U/L
ALT	162	0-49 U/L
Hemoglobin	6.5	14-18 g/dL
Hematocrit	19.3	38%-49%
WBC	31.6	3.4-10.8 x 10⁹/L
Neutrophil count	29.39	2.2-4.8 x 10⁹/L
Lymphocyte count	0.95	1.3-2.9 x 10⁹/L
Monocyte count	1.26	0.3-0.8 x 10⁹/L
Eosinophil count	2.5	0.0-0.7 x 10⁹/L
Fibrinogen	243	194-496 mg/dL
Proteinuria	2+	Negative
Hematuria	3+	Negative
Urine RBCs	80	0-2/HPF
Rheumatoid factor	51	0-29 IU/mL
Glomerular basement membrane antibody	<0.2	<1 AI
Myeloperoxidase antibody	<1	<1.0 AI
Proteinase 3 antibody	384.1	<0.1 AI
C3 complement	75	87-200 mg/dL
C4 complement	<8	19-52 mg/dL
C-ANCA	Positive	Negative
HIV antigen/antibody 4th generation	Negative	Negative
ANA	Negative	Negative
DsDNA antibody	<1	4-10 IU/mL
Anti-Jo-1 antibody	Negative	Negative
Ribosomal protein antibody	Negative	Negative
Scleroderma SCL-70 antibody	Negative	Negative
Sjogren SS-A/B antibody	Negative	Negative
Anti-smooth muscle antibody	<20	<20 U
Anti-Smith antibody	Negative	Negative
Anti-SM/ribonuclear protein (RNP) antibody	Negative	Negative
Anti-centromere antibody	Negative	Negative
Anti-U1-RNP antibody	Negative	Negative
Chromatin nucleosomal antibody	Negative	Negative
Hepatitis panel	Negative	Negative

Figure 1B of CT sinus showing a 1-cm polyp in the inferior aspect of the left maxillary sinus. This led to initial suspicion of EGPA. He was treated with pulsed-dose intravenous methylprednisolone 1000 mg for five days, then transitioned to 80 mg IV daily. During the course of admission, he developed hemoptysis, for which a CT chest shown in Figure 1A revealed bilateral pleural effusions, which were secondary to renal failure.

**Figure 1:**
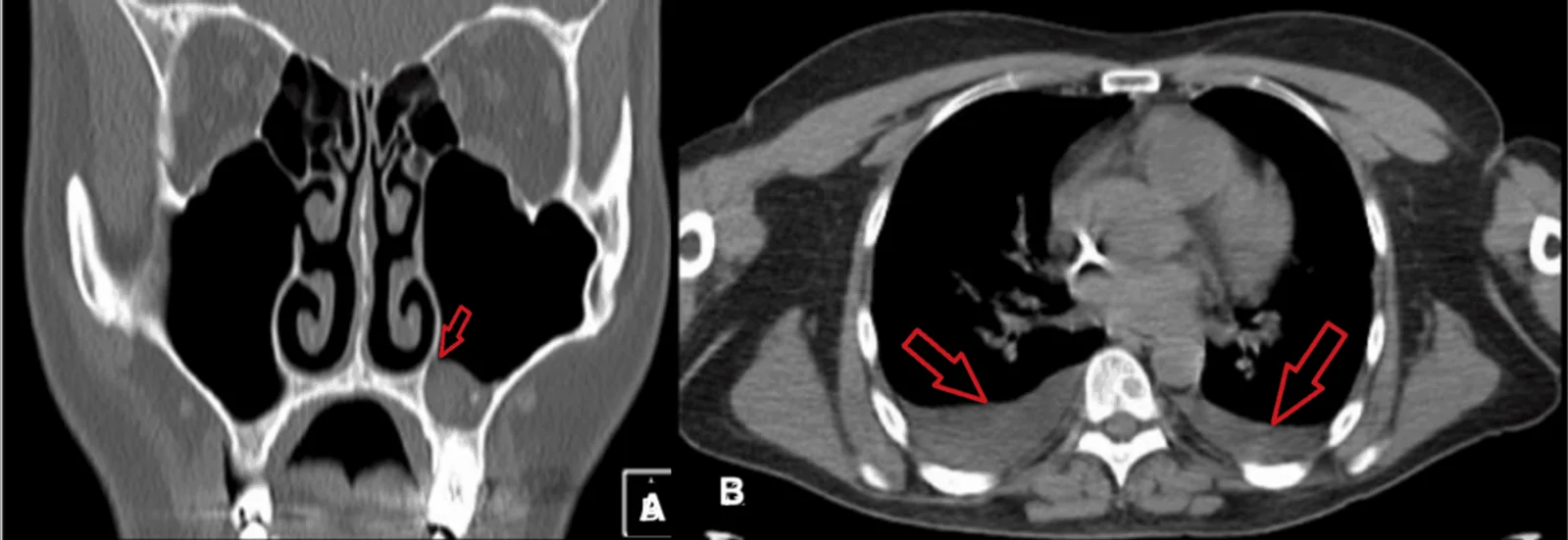
(A) CT sinus with a 1-cm polyp in the inferior aspect of the left maxillary sinus. (B) CT chest showing bilateral pleural effusions. CT of the sinus in Figure 1A shows a 1-cm polyp in the inferior portion of the left maxillary sinus, a characteristic finding in EGPA. CT of the chest in Figure 1B shows bilateral pleural effusions, worse on the right. This was considered secondary to renal failure. EGPA, eosinophilic granulomatosis with polyangiitis.

He underwent plasmapheresis (five sessions) and received three of four planned rituximab (375 mg/m^2^ doses) while on admission (receiving the 4th dose following discharge). Renal biopsy later confirmed focal necrotizing and crescentic glomerulonephritis with an eosinophil-rich infiltrate, leading to a final diagnosis of EGPA, as shown in Figures 2, 3, showing immunofluorescence with a pauci-immune pattern. Given low complement levels, other considerations were cryoglobulinemic vasculitis and lupus nephritis. However, renal biopsy led to the diagnosis of EGPA.

**Figure 2:**
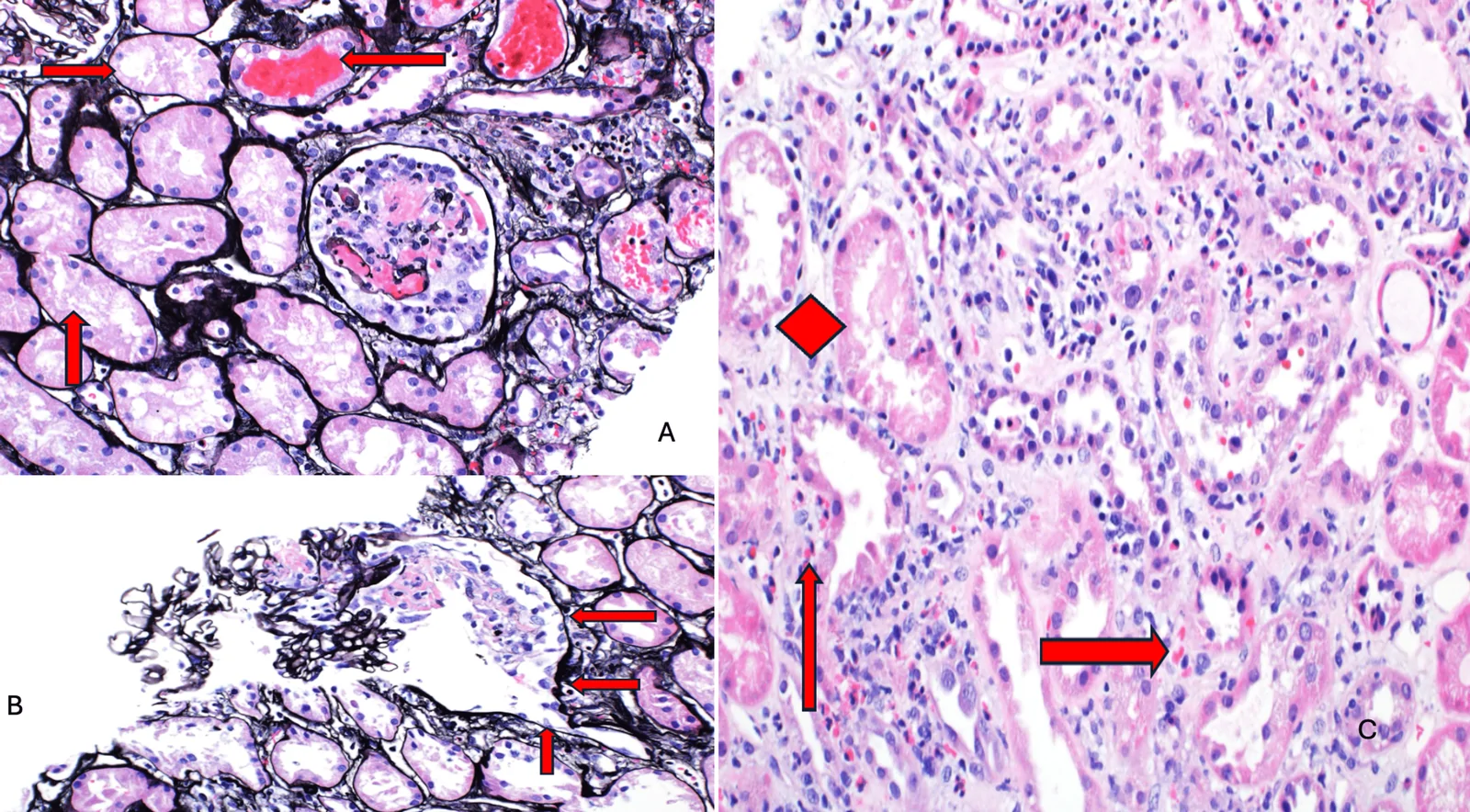
Renal biopsy images. (A and B) Periodic acid-methenamine silver stain showing focal necrotizing and crescentic glomerulonephritis. (C) Hematoxylin and eosin stain showing an eosinophil-rich interstitial inflammatory infiltrate. Image A: Silver stain. Red arrows highlight tubular cell disruption with evidence of necrotic infiltrates. Image B: Silver stain image with red arrows that show crescentic-appearing glomeruli with disruption of the basement membrane. Image C: Hematoxylin and eosin stain; red arrows show eosinophil-rich interstitial infiltrates; the diamond arrow highlights evidence of interstitial edema and inflammatory cells.

**Figure 3:**
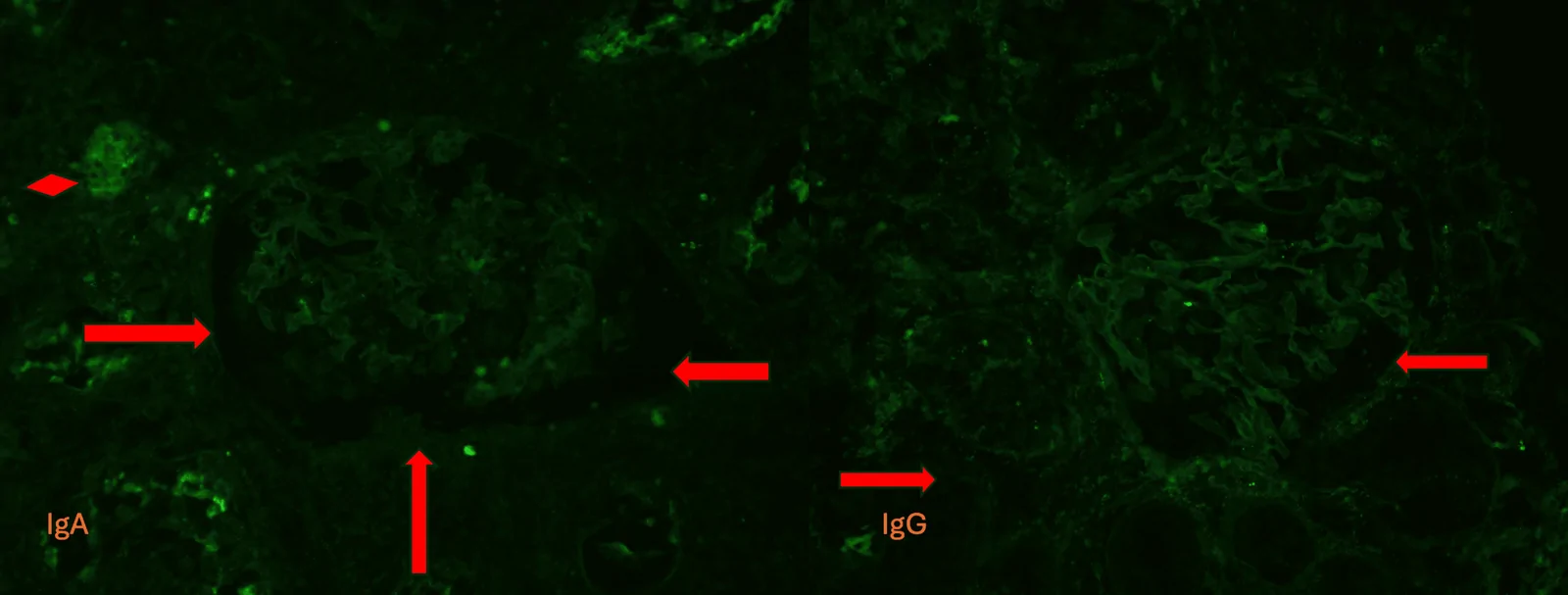
Immunofluorescence staining with a pauci-immune pattern with no significant staining for immunoglobulin Image IgA shows a central glomerulus with no significant granular, linear, or mesangial deposition of immunoglobulins, which is the hallmark of a pauci-immune process. Diamond shows small, scattered bright dots (likely autofluorescence or non-specific protein trapping) in the periglomerular tissue and within the tuft. These do not represent true immune deposition. Red arrows in the IgG image indicate negative granular/linear Ig staining, with no mesangial deposits.

Eosinophil count continued to improve on admission following initiation of treatment. It had normalized at discharge follow-up. The patient's renal function improved, with creatinine at 2.0 mg/dL at discharge. He was discharged on prednisone 60 mg daily, which he completed as a taper regimen. He also completed rituximab therapy. He has had renal recovery, with creatinine back to normal (1.07), with no requirement for dialysis after discharge. Within three months of discharge, eosinophil count was normal; creatinine subsequently ranged from 1.0 to 1.1 at nephrology follow-ups. Post-treatment ANCA screen was negative, with myeloperoxidase (MPO)/PR3 antibodies <1.0 AI.

## Discussion

EGPA is a rare systemic vasculitis of unclear etiology that classically presents with bronchial asthma, eosinophilia, and necrotizing vasculitis [4]. In contrast to GPA, which presents with destructive sinonasal lesions, pulmonary nodules, and pauci-immune glomerulonephritis [1], EGPA is typically associated with PR3 antibodies, whereas GPA is linked to MPO antibodies in about 40% of cases, with PR3 positivity being rare [1,5].

Overlap syndromes between EGPA and GPA have been described, with one review of 15 cases reporting 80% in females, universal lung involvement, and renal involvement in over 50% [5]. Our patient presented primarily with renal involvement, PR3 positivity, and eosinophilic vasculitis, which was atypical for EGPA. Initially, GPA was suspected due to PR3 antibody positivity. However, based on the 2022 ACR/EULAR scoring system [3], he met criteria for EGPA with a score of 7 points. This was due to the presence of nasal polyp (+3 points), blood eosinophil ≥1 x 10^9^ (5 points), demonstration of eosinophil-predominant inflammation on biopsy (2 points), neuropathy (1 point), the penalty for PR3-ANCA positivity (-3 points), and presence of hematuria (-1 point). Per the 2022 ACR/EULAR GPA criteria, the patient had +5 points for PR3-ANCA positivity. However, the patient had -4 points for eosinophils >1,000/uL. Thus, the total score was -1, which did not meet the ≥5 points required for GPA classification.

Overlap syndromes between EGPA and GPA have been described, with one review of 15 cases reporting 80% in females and universal lung involvement [6]. Patients with overlapping features often present with hallmark EGPA signs (such as severe asthma, nasal polyps, and tissue eosinophilia) and classic GPA markers (such as positive anti-PR3 antibodies, alveolar hemorrhage, and necrotizing granulomas) [7]. Our patient demonstrated an unusual hybrid overlap phenotype, highlighting why strict criteria can be difficult to satisfy without an authentic overlap in clinical presentation. Treatment of EGPA involves glucocorticoids and immunosuppressants, with cyclophosphamide for severe cases. The choice of rituximab over cyclophosphamide for our patient was influenced by PR3-ANCA positivity. The decision on plasmapheresis preceded the PEXIVAS trial results. It was also driven by persistence of bleeding episodes. Maintenance options include methotrexate, azathioprine, or mycophenolate [1]. Rituximab is usually preferred for relapses and refractory cases [1]. Emerging therapies such as interleukin-5 inhibitors (mepolizumab, benralizumab, and resilumab) show promise in EGPA, particularly in eosinophilic asthma, though data remain limited regarding the role in remission induction or refractory disease management [8].

## Conclusions

PR3-positive EGPA is a rare and diagnostically challenging form of ANCA-associated vasculitis that can significantly overlap with GPA. Our patient, without a history of asthma, presented with recurrent sinusitis and epistaxis and positive PR3-ANCA serology. This initially raised strong suspicion for GPA. However, a comprehensive evaluation, including renal biopsy, ultimately established the diagnosis of EGPA. This case underscores the critical role of renal biopsy and the importance of integrating clinical, serologic, and histopathologic findings when evaluating atypical presentations of ANCA-associated vasculitis. Treatment typically includes glucocorticoids, immunosuppressive agents, and biologic therapies. Our patient received rituximab, plasmapheresis, and prednisone with clinical improvement. We would recommend further research to optimize the diagnosis of PR3-positive EGPA, especially in the absence of asthma or clinically defining clues, and guidance on management choices in patients with overlap phenotypes.
